# The Impact of Post-Stroke Depressive Symptoms on Cognitive Performance in Women and in Men: A 4 Month Prospective Study

**DOI:** 10.3390/life13071554

**Published:** 2023-07-13

**Authors:** Matildes F. M. Sobreiro, Luisa Terroni, Valeri Delgado Guajardo, Patricia Ferreira Mattos, Claudia da Costa Leite, Edson Amaro, Gisela Tinone, Dan V. Iosifescu, Renerio Fraguas

**Affiliations:** 1 Grupo de Interconsultas, Departamento e Instituto de Psiquiatria do Hospital das Clínicas, Faculdade de Medicina da Universidade de São Paulo, Rua Dr. Ovídio Pires de Campos, 785, São Paulo CEP 05403-903, Brazil; 2 Departamento de Radiologia do Hospital das Clinicas, Faculdade de Medicina da Universidade de São Paulo, São Paulo 05403-000, Brazil; 3 Departamento de Neurologia, Instituto Central do Hospital das Clinicas, Faculdade de Medicina da Universidade de São Paulo, São Paulo 05403-000, Brazil; 4 New York University School of Medicine and Nathan Kline Institute, New York, NY 10003, USA; 5 Laboratório de Investigações Médicas, LIM 21, Departamento e Instituto de Psiquiatria do Hospital das Clínicas, Faculdade de Medicina da Universidade de São Paulo, Rua Dr. Ovídio Pires de Campos, 785, São Paulo CEP 05403-903, Brazil; 6 Divisão de Psiquiatria e Psicologia, Hospital Universitário, Universidade de São Paulo, São Paulo 05403-903, Brazil

**Keywords:** depression, depressive symptoms, ischemic stroke, neurocognitive, gender, sex differences, neuropsychological

## Abstract

**Background**: Depressive symptoms have been associated with cognitive impairment after stroke, and women may be specifically affected. **Objective**: The aim of this study was to investigate gender-specific characteristics in the relationship between changes in depression severity and changes in cognitive performance after stroke. **Methods**: We prospectively evaluated 73 patients without a previous history of depression in the first and fourth months after a first ischemic stroke. The severity of depressive symptoms was assessed using the 31-item version of the Hamilton Rating Scale for Depression, and executive function, attention, working memory, and verbal fluency were assessed using a neuropsychological battery. **Results**: We included 46 (63.0%) men and 27 (36.9%) women, with mean ages of 55.2 (SD ± 15.1) and 46.8 (SD ± 14.7) years, respectively. We found significant improvement in the digit span forward and Stroop dots from month 1 to month 4 post stroke for both men and women. Women, but not men, presented a correlation between changes in phonemic verbal fluency and changes in the 31-item version of the Hamilton Rating Scale for Depression scores. Improvement in depression was correlated with improvement in verbal fluency, and worsening in depression was correlated with worsening in verbal fluency. **Conclusions**: Our results suggest that women might be more vulnerable to the relationship between depressive symptoms and cognitive performance, and improvement of depression may be necessary for women’s improvement in phonemic verbal fluency from the first to the fourth month after a stroke. We did not adjust the results for multiple comparisons. Thus, our findings might be considered preliminary, and confirmatory studies, also focusing on specific characteristics of women that could explain these differences, are warranted.

## 1. Introduction

Gender inequalities in stroke outcomes have put women stroke survivors at a disadvantage [1]. While stroke incidence has historically been higher in men [2], recent studies focusing on young adults have shown a similar stroke incidence for ages 35 to 45 years and an even higher incidence (incidence rate ratio of 1.44) in women for ages ≤35 years [3]. Although women may not experience a clear disadvantage in terms of stroke incidence compared to men, evidence from the literature consistently shows that women have worse outcomes after stroke. Stroke in women has been associated with a greater change in consciousness/mental status, a higher incidence of coma/stupor [4], and a higher risk of being severe, according to the National Institutes of Health Stroke Scale (NIHSS), compared to men [5]. After a stroke, women have been reported to have a poorer health-related quality of life in physical and mental domains, worse performance in daily living activities, and higher levels of anxiety than men [6]. Depression and impairment in cognitive functions significantly impair daily living activities [7] and are among the most frequent unmet needs after stroke [8].

Mild cognitive impairment has been reported in 17% to 92% of patients 3 months after stroke [9]. Commonly affected functions by a stroke include attention, spatial ability, language, and executive ability [10]. In addition to brain location and lesion size [11,12], biomarkers indicate that neuropsychological impairment after a stroke is related to inflammatory factors, growth factors (i.e., insulin-like growth factor 1 and brain-derived neurotrophic factor), oxidative damage, and genetic factors [13]. Studies have reported differences in cognitive performance between men and women after stroke. Although not consistently significant in all studies, post-stroke cognitive deficits, particularly during the early post-stroke period, tend to be more frequent in women than in men [6,14]. Sociodemographic pre-stroke characteristics including being widowed, older age, and lower educational attainment may contribute to a worse cognitive outcome after stroke among women [15]. However, the specific impact of other factors, such as the relevance of depression have not been properly investigated.

Depression occurs in around 30% of stroke survivors [16,17,18]. Although not consistent across all studies [18,19], depression negatively impacts post-stroke patients. It has been associated with reduced quality of life, poorer prognosis, and increased mortality [20]. It can also restrict social contacts and cause dissatisfaction with life partners and friends [21]. Post-stroke depression has been related to lesions in the frontal area [22], particularly in the limbic–cortical–striatal–pallidal–thalamic (LCSPT) circuit [23] and with altered functional connectivity in the left inferior parietal gyrus, left orbital part of the inferior frontal gyrus, and left angular gyrus [24]. Large-scale network disruptions following stroke have also been associated with depressive features even without lesions in the dorsolateral prefrontal cortex (DLPFC) [25]. Differences between men and women have also been reported for post-stroke depression. Most studies have reported a higher prevalence of post-stroke depression in women compared to men [20,26], particularly within the first months after a stroke [27]. There is also a trend indicating poorer outcomes associated with depression in women compared to men [6]. For women, but not men, depressive status after stroke has been identified as one of the factors determining lower recovery of daily living activities and physical functioning [28].

There is an association between cognitive functions and depression after a stroke. Depression has been associated with cognitive impairment 6 months after stroke [29]. In older adults, the emergence of depression and its chronicity have been reported to be significant predictors of worse post-stroke cognition [30]. Furthermore, specific symptoms, such as apathy, have been shown to be associated with greater impairment in executive function, memory, and global cognition [31]. On the basis of the aforementioned findings, it is reasonable to hypothesize that the progression of depressive symptom severity after a stroke influences the progression of neuropsychological performance, and that this influence has specificities in men and women. Therefore, this study aimed to investigate the gender-specific relationship between changes in severity of depressive symptoms and changes in cognitive performance after a stroke.

## 2. Methods

### 2.1. Sample

**Figure 1:**
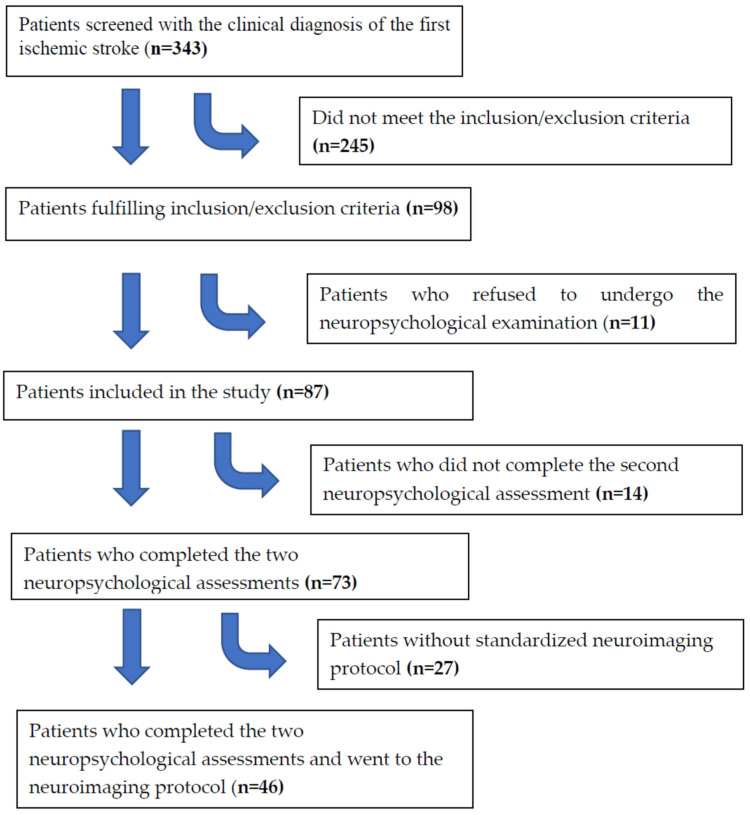
Flowchart of patients according to inclusion/exclusion criteria.

We included patients consecutively admitted to a university hospital with a diagnosis of ischemic stroke. Protocol details were described previously [23]. Briefly, the stroke diagnosis was made according to World Health Organization criteria [32] and confirmed by magnetic resonance neuroimaging (MRI). The MRIs were acquired in general within 2 weeks after the stroke. To be included, the patient had to be 18 years old or older, be able to understand and provide signed informed consent, and not have a previous ischemic stroke. We excluded patients with a current depressive episode starting before the stroke, as well as patients with a previous history of major depressive disorder (MDD). Of the 343 screened patients with a clinical diagnosis of ischemic stroke, 245 were excluded because they did not meet the inclusion/exclusion criteria, and 73 patients completed the two assessments (see flowchart in Figure 1). Of the 73 patients, 50 had complete MRI for the analyses of lesion in cortex studied. The Stroop test [33] was included in the protocol during the study, and only 36 patients undertook it at the two timepoints. The hospital ethics committee approved the study protocol. Data were collected between 2002 and 2008.

### 2.2. Procedures

Patients were evaluated at two timepoints by a neuropsychologist and by a psychiatrist: initially (T1) within the first month after the stroke (5–25 days after the stroke) and then in the fourth month after the stroke (80–110 days after stroke) (T2).

### 2.3. Assessment of Depressive Symptoms

The severity of depressive symptoms was assessed by a psychiatrist using the 31-item version of the Hamilton Rating Scale for Depression (HAM-D-31) [34,35] at the two timepoints. The HAM-D-31 has the advantage of assessing a more extensive number of depressive symptoms, including reverse ones such as increased appetite, weight gain, three hypersomnia items, and two additional retardation items. Each item is rated on a scale of 0–4 or 0–2, with higher scores indicating greater severity of symptoms.

### 2.4. Neuropsychological Assessment

A neuropsychologist made the neuropsychological assessment at the two timepoints. The protocol included the evaluation of attention and working memory with the forward and backward digit span task, a subtest of the Revised Wechsler Adult Intelligence Scale [36], Brazilian version [37]. The span digit subtest is composed of two tasks that are applied independently of each other (forward and backward). In both tasks, the examiner reads a series of number sequences aloud and the respondent should repeat the numerical sequence in the same order presented. For backward, the patient should repeat the numerical sequence in the opposite order (backward) to that presented by the examiner. The examiner starts with a sequence of two digits and continues with longer sequences until the participant is unable to correctly repeat the digits for two trials at the same length. For each numerical sequence correctly completed, one point is assigned. Executive function was evaluated with the verbal fluency test (phonemic test), with words beginning with the letters F, A, and S (FAS test) [38] and with the Victoria version [39] of the Stroop color and word test [33]. In the FAS test, patients are asked to speak all the words they can remember that begin with each of the F, A, and S letters during the time of 1 min. They must not include proper names or derived words. As a result, the total words produced in the three categories (F, A, and S) were computed, excluding errors and repeated words. In the Stroop version we used, three conditions were assessed: in the first (Stroop dots), the patient was asked to say the color of the dot; in the second (Stroop word), the patient was asked to say the color in which neutral words are written; in the third (Stroop color), the patient was asked to say the color in which words are written. However, in this last condition, the words were the names of a color written in a noncorresponding color (e.g., the word yellow written in blue color). We calculated the Stroop interference scores dividing the Stroop color (time was adjusted by the number of correct answers) by Stroop dots (time was adjusted by the number of correct answers) [40].

### 2.5. Assessment of Functional Status

The National Institutes of Health Stroke Scale (NIHSS, range 0–42) [41] was used to quantitatively assess the functional status. Higher scores represent greater severity of the neurological sequelae of stroke. A neurologist used the NIHSS within the first month after the stroke (5–25 days after the stroke—T1) and then in the fourth month after the stroke (80–110 days after stroke—T2).

### 2.6. MRI Methods and Assessment

The MRIs were acquired in general within 2 weeks after stroke (9.34 ± 6.87 days; range 1–43 days), and details of the protocol were published previously [23]. The images were acquired using a 1.5 T system (GE-Horizon LX). The imaging protocol included axial spoiled gradient recalled acquisition in steady state (SPGR, TR 27 ms; flip angle 45°; voxel size 0.94 × 0.94 × 1.5 mm), axial fluid-attenuated inversion recovery (FLAIR, TR 133 ms; TE 8400 ms; TI 2100 ms; voxel size 0.94 × 0.94 × 5 mm), axial diffusion-weighted image (TR 8000 ms; b value 1000 s/mm^2^; voxel size 1.8 × 1.8 × 5 mm), and T2-weighted fast spin echo (TR 4500 ms; TE 100–120 ms; voxel size 0.94 × 0.94 × 5 mm). All images were acquired in the bicommissural plane. Lesion location and volume quantification were determined using a semi-automated method. Initially, SPGR and axial FLAIR acquisitions were both normalized to the Montreal Neurological Institute template [42] using linear transformation with 12 degrees of freedom and 15 nonlinear interactions implemented in Statistical Parametric Mapping (SPM5, Wellcome Trust for Neuroimaging, London, UK https://www.fil.ion.ucl.ac.uk/spm/ (accessed in 2008)) [43], on the basis of coordinates referenced in the Talairach and Tournoux Atlas [44]. During this process, all images were sampled to 2.3 × 2.3 × 2.6 mm. Lesion delineation was performed by a trained psychiatrist (LT) using a mouse device to trace the ischemic lesion and analyzing all slices of each FLAIR image using MRIcro Software version 1.39 (http://www.sph.sc.edu/comd/rorden/mricro.html)/ (accessed in 2008) and currently accessible on https://people.cas.sc.edu/rorden/mricro [45]. All lesions’ delineations of each patient were reviewed by a neuroradiologist (EAJ), blinded for clinical data and psychiatric diagnoses. Lesion volume was expressed as the mean and standard deviation of voxels in mm^3^ (FLAIR voxel size 2.3 × 2.3 × 2.6 mm). The volume of the lesion in the prefrontal cortex areas was estimated by the following formulas: orbitofrontal cortex = BA10 (Brodmann area 10) + BA11 + BA12 + BA13 + BA47; medial prefrontal cortex = BA10 + BA11 + BA13 + BA14 + BA24 + BA25 + BA32 + BA47; dorsolateral prefrontal cortex = BA9 and total volume by summing the 48 Brodmann areas.

### 2.7. Analysis

We analyzed depressive symptom severity and cognitive performance in the first (T1) and the fourth (T2) months after the stroke in the total sample and in gender-defined subsamples. Descriptive analyses included mean, standard deviation, and absolute and relative frequencies for quantitative and qualitative variables, respectively. We used neuroimaging data to investigate specificities in stroke location/volume in women and men. Student’s *t*-test or the Mann–Whitney U-test was used for gender comparison, when the normality distribution was satisfied or not, respectively. To investigate differences between T1 and T2 measurements by gender, we applied two-way repeated-measures ANOVA, considering the Holms pairwise correction for post hoc tests. Furthermore, we used Student’s *t*-test for dependent samples or the Wilcoxon rank test for time comparison. We employed Spearman correlation coefficients to investigate the relationship between changes in the severity of depressive symptoms and changes in the neuropsychological performance; results were adjusted for age but not for multiple comparisons, considering the exploratory nature of this study. To investigate specificities of this relationship in men and women, the main objective of our study, we split that analysis by gender. The changes in the variables were calculated through the difference at the two times measured (T2–T1). The variables distributions were verified using the Shapiro–Wilk test. All statistical tests were based on two-tailed significance at the 5% level (α = 0.05). We used the JAMOVI computer software and R Language and environment for statistical computing. To perform the analysis, we used the jamovi project (2022) (Version 2.3) [computer software], retrieved from https://www.jamovi.org (accessed in April 2023) and the R Core Team (2021; R: A Language and environment for statistical computing. (Version 4.2.3)) [Computer software], retrieved from https://cran.r-project.org (accessed in April 2023) (R packages were retrieved from MRAN snapshot 1 January 2022).

## 3. Results

### 3.1. Sample, and Men’s and Women’s Characteristics

We prospectively included 73 patients after their first ischemic stroke, without previous history of MDD; 46 (63.0%) were men and 27 (36.9%) were women. Women were significantly younger than men (*p* = 0.0245) with mean ages of 55.2 (SD ± 15.1) and 46.8 (SD ± 14.8) years, respectively. No other sociodemographic characteristics were different between genders. Most men and women were married, n = 33 (73.3%) and n = 13 (48.2%), respectively, and were working before the stroke, n = 29 (64.4%) and n = 16 (59.3%), respectively. On average, men had 6.5 (±3.8) and women had 7.7 (±4.2) years of education. Stroke lesion on the left occurred in 17 (63.0%) women and in 26 (59.1%) men (*p* = 0.746); and stroke lesion on the right side occurred in 10 (37%) women and in 20 (45.5%) men (*p* = 0.486). Demographics, clinical characteristics, and cognitive performance for the total sample and gender-based subsamples are reported in Table 1. Gender was not associated with volume of the total Brodmann area or with any analyzed prefrontal cortical areas.

### 3.2. Evolution from the First to the Fourth Month after Stroke

Patients underwent neuropsychological evaluations at two timepoints, at a mean of 12 (SD = ±3.8, range = 6–23 days) and a mean of 91.6 days (SD = 5.4, range = 83–108 days) after the stroke. No significant decrease in cognitive performance was found on any neuropsychological test. The stroke scale NIHSS exhibited a significant reduction (*p* < 0.001). Severity of depressive symptoms did not significantly change from T1 to T2.

### 3.3. Specificities of Men and Women in the Evolution

There was no significant time × gender interaction effect for any of the neuropsychological variables (*p* > 0.05; see Table 2). From the first to the fourth month after the stroke, women and men showed an improvement in digit span forward and Stroop dots (Supplementary Materials Table S1). Although the time × gender interaction was nonsignificant, we performed an exploratory analysis splitting the sample by gender; differences from T1 to T2 in some neuropsychological tests in women and others in men are presented in the Supplementary Materials, as recommended, as preliminary analyses for future studies adequately powered to detect gender differences [46]. The severity of depressive symptoms was higher in women than men in T2 (also in T1; *p* < 0.031. Table 2). The improvement in NIHSS was independent of being man or woman. We reached a statistical power of 73% for our sample to detect a difference between T1 and T2 measurements by gender.

### 3.4. Correlation of Changes in Severity of Depression with Changes in Cognitive Performance and with Functional Status: Specificities of Women and Men

**Figure 2:**
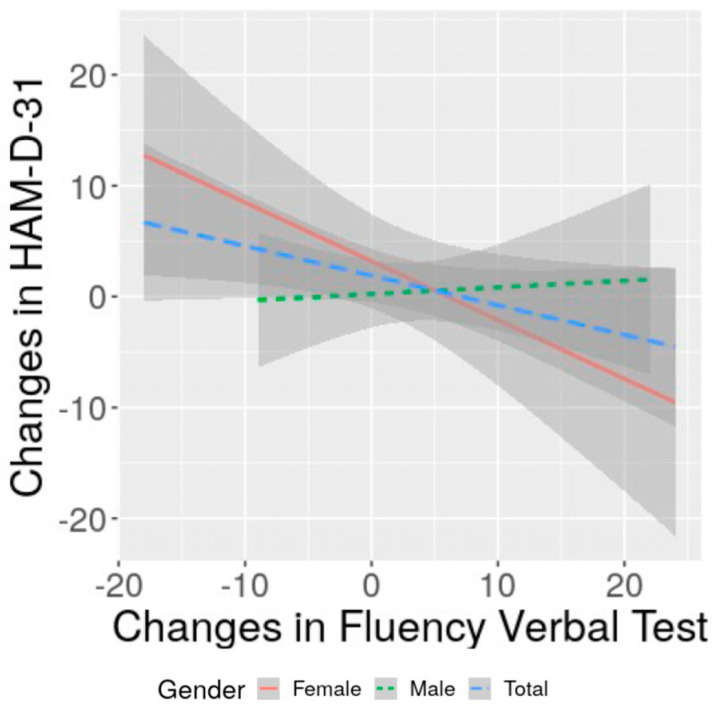
Correlation between changes in phonemic verbal fluency test with changes in HAM-D-31.

The analysis investigating the correlation of cognitive changes with changes in depressive symptoms severity showed no association (*p* > 0.05) for the total sample. Splitting the analysis by gender and adjusting for age revealed that only women presented an inverse significant (r = −0.4, *p* = 0.039) correlation between changes in verbal fluency and changes in HAM-D-31 scores (Table 3; Figure 2). Women also presented a significant direct correlation between changes in verbal fluency and changes in NIHSS adjusted for age (rho = 0.450, *p* = 0.021). For men, no correlation was found between changes in the depressive symptom severity with changes in their cognitive performance or with changes in NIHSS. Age was statistically directly associated with changes in digit span backward for women (r = 0.55, *p* = 0.003), but with no other changes in neuropsychological tests. Age was not correlated with changes in verbal fluency for total sample (*p* = 0.616), for women (*p* = 0.483) or for men (*p* = 0.845).

## 4. Discussion

In this prospective study involving 73 post-stroke patients, consisting of 46 (63.0%) men and 27 (37.0%) women, we found that changes in the severity of depressive symptoms from the first to the fourth month after stroke were inversely correlated with changes in phonemic verbal fluency among women but not men. This correlation suggested that more favorable changes in the severity of depressive symptoms led to more favorable changes in neuropsychological performance, while more adverse changes in the severity of depressive symptoms led to more adverse changes in neuropsychological performance.

Various studies have proposed that depression leads to cognitive impairment [47]. In line with this view, our results suggest that changes in depression severity in women would lead to changes in phonemic verbal fluency. Studies conducted on non-stroke samples have generated inconclusive results regarding the cognitive impairments that may improve with the amelioration of depression and whether they should be considered state or trait markers. A meta-analysis including bipolar patients demonstrated a greater decline in phonemic fluency measures during depressed episodes compared to euthymic patients [48]. This suggests that phonemic fluency could serve as a state marker of depression. In contrast, verbal learning and memory remained impaired during the euthymic phase, indicating their potential as trait markers of depression, and reflecting a genetic predisposition to the illness [48]. However, other studies have found that the progression of depression severity correlated with semantic fluency rather than phonemic fluency [49], and phonemic fluency did not ameliorate with a decrease in depression severity in women [50]. With respect to gender, the literature is conflicting in communities of older adults. One study found that the presence of depressive symptoms at baseline was associated with a decline in verbal fluency performance during longitudinal follow-up in female but not male participants [51], whereas another study showed that depression at baseline was predictive of cognitive decline in males but not females [52]. These discrepancies in the literature have not been fully elucidated or explained. It has been proposed that deficits in phonemic and semantic fluency tests among patients with depression may not solely indicate executive dysfunction, but instead, a more generalized impairment such as cognitive slowing [53]. It is possible that depression may impact various executive functions in distinct ways [54]. It has also been suggested that the level of effort exerted by individuals to compensate for phonemic fluency impairment in depression might interfere with test performance, leading to discrepancies in results [55]. On the basis of those findings from non-stroke patients, it is possible that post-stroke depression also impacts different cognitive functions in discordant ways. In our sample, it is possible that changes in depression severity could have affected the effort to compensate for phonemic verbal fluency in women and, consequently, their performance, but may not have affected men’s effort.

Considering stroke patients and the view that depression leads to cognitive impairment, post-stroke depression has been associated with worse performance in cognitive functions, including inhibition/switching errors [46], memory [56,57], reduced psychomotor/cognitive speed [57,58], nonverbal problem solving, and attention [57]. Considering the impact of gender, women who have experienced lacunar strokes have been found to have cognitive impairment more frequently than men, and this association has been shown to be dependent on the occurrence of depression and severity of white matter hyperintensities in women but not in men [59]. In line with that, with regard to specificity, our results suggest that, after stroke, there may be a correlation between changes over time in depressive symptom severity and changes in phonemic verbal fluency in women, but not in men. This finding, specifically in women, might require specific clinical attention (i.e., treatment of depression). It is possible that, for women, an improvement in depression is necessary to allow an improvement in phonemic verbal fluency. Such correlation implies that, particularly for women, early detection and adequate treatment of depression may be crucial not only to decrease the suffering caused by depression, but also to ensure a better neuropsychological improvement, particularly in phonemic verbal fluency.

Considering the treatment of post-stroke depression, it has been shown that treatment with selective serotonin reuptake inhibitors is associated with higher cognitive function compared to placebo [60]. Additionally, successful treatment of depression has promoted cognitive recovery in stroke victims [61], and remission of depression may be fundamental for improvement in cognitive impairment [62]. Antidepressant treatment of post-stroke depression has been particularly effective for women [63]. Notably, depression with executive dysfunction tends to have a more chronic course of depressive symptoms than depression without executive dysfunction and requires special attention [62]. Furthermore, both woman and depression have been related to worse quality of life after stroke [64].

The relationship between cognitive performance and depression after stroke Is not fully understood. On one hand, depression could lead to cognitive impairment as we discussed above. Alternatively, with some exceptions [65,66], studies have proposed that cognitive impairment could be the cause, with depression as the consequence [65,67]. One possible explanation is that cognitive improvement associated with lower emotional distress [68] could reduce depression morbidity. Another possibility is that both depression and cognitive deficits are caused by common biological factors, such as serotonin or inflammatory changes, or changes in brain connectivity. Decreases in serotonin levels from baseline to after rehabilitation have been correlated with worsening in cognitive functions, as indicated by changes in the Rey’s figure, and the Tower of London test [69]. The altered inflammatory response by astrocytes has also been proposed to explain depression and cognitive impairment after stroke [70]. It is also possible that disruption of connections in the cognitive and limbic systems [71] caused by the vascular accident could explain the relationship between post-stroke depression and cognitive impairment. Specifically, post-stroke depression and executive dysfunction could result from functional overconnectivity between the default mode and salience/cognitive control networks and reduced cross-hemispheric frontoparietal functional connectivity [16]. Lastly, it is theoretically possible that post-stroke depression and cognitive impairment are independent phenomena for some patients.

Explaining the differences between men and women regarding cognitive performance, depression, and their relationship after stroke is more complex. These differences may stem from predictors of post-stroke depression. Reported predictors for men include exercise habits and fibrinogen levels, while, for women, predictors were brain-derived neurotrophic factor (BDNF), magnesium, and free T3 [72]. Gender-specific differences in stroke topography may also contribute to these distinctions. A pattern of left-hemispheric posterior circulation brain regions, including the left hippocampus, precuneus, fusiform and lingual gyrus, occipital pole and latero-occipital cortex, has been found to have a substantially higher relevance in explaining functional outcomes in women compared to men [73]. In our sample, we did not find a relationship between gender and the volume of the lesion in the cortex defined by Brodmann areas.

Of note, we found cognitive improvement from the first to the fourth month after the first ischemic stroke for both men and women. This favorable evolution could be attributed, in part, to the relatively young age of our sample, with a mean age of 46 years for women and 55 years for men. The effect of practice may also influence the performance improvement in the reapplication of neuropsychological tests. It tends to be prominent when the test is repeated in a short interval such as one week [74]; after 7 weeks, the practical effect is considered to be of small size [75]. In our study, the interval between the neuropsychological tests was 3 months; thus, any practice effect should be minimal. Our patients diagnosed with depression were offered treatment, which might have mitigated possible cognitive deficits associated with depression. Notwithstanding this relatively favorable scenario in our sample, it has been reported that 35.4% of patients still showed cognitive impairment at 12 months after stroke [76], which enhances the necessity of early detection and treatment of both depression and cognitive impairment.

Our study had some limitations to be considered. It was conducted at a single site, a university hospital, with a relatively small sample size (n = 73, including 37 women). Further studies are necessary to confirm and generalize our results to other samples. We investigated the relationship between changes in cognitive performance and changes in the severity of depressive symptoms of all patients, not only in those with depression. We adopted this approach considering that symptoms of depression in post-stroke patients without the diagnosis of major depression might still have a negative impact and require attention [77]. Women in our sample were younger and had fewer years of education compared to men, and older age and lower education were associated with lower cognition scores. Thus, age and years of education could be confounders of our results. However, age and years of education were not related to changes in verbal fluency, and the relationship between changes in verbal fluency and changes in severity of depression remained statistically significant after adjusting for age in women. We enrolled only patients after first stroke to reduce sample variability and the effect of previous strokes, which resulted in a sample of relatively young patients. Therefore, these results may not be applicable to older post-stroke patients. In fact, for older post-stroke patients, depression could be more deleterious for cognition in men than in women [78]. We also did not investigate the influence of health status and stroke complications [79] including menopause, level of fatigue, sleep problems and disorders, fever or recent infection, seizures, hemineglect, hyperglycemia, and other cardiac/respiratory/hepatic medical conditions, which could also play a role in the severity of depression and neuropsychological performance, as well as their changes and relationship. We did not formulate a specific hypothesis regarding the correlation between changes in depression severity and changes in a particular neuropsychological test, nor did we incorporate a correction for multiple comparisons in our analysis. Consequently, our findings should be deemed preliminary in nature. Strengths of our study include its prospective design with serial assessments, the use of a neuropsychological test battery administered by a neuropsychologist, and the assessment of depressive severity by psychiatrists using the HAM-D-31 comprehensive depression scale.

## 5. Conclusions

In conclusion, our prospective study with 73 post-stroke patients suggests that women may be more vulnerable to the relationship between changes in depressive symptoms severity and changes in neuropsychological performance from the first to the fourth month after stroke. For women, changes in phonemic verbal fluency may be associated with changes in depressive symptom severity. Our findings suggest the need for early depression detection and treatment for post-stroke women, aiming to improve cognitive performance in addition to diminishing the suffering and ameliorating the negative impact on quality of life caused by depression. We did not adjust for multiple comparisons, and our findings should be considered preliminary. Confirmatory studies, investigating specific underlying factors that could explain such differences, are warranted.

## Figures and Tables

**Table 1:** Demographic, clinical characteristics, and neuropsychological performance 1 and 4 months after a first ischemic stroke: Total sample and split by gender.

	T1									T2								
Variables	Total (N = 73)			Female (N = 27)			Male (N = 46)			Total (N = 73)			Female (N = 27)			Male (N = 46)		
	N	M	SD	N	M	SD	N	M	SD	N	M	SD	N	M	SD	N	M	SD
Age (years)	73	52.1	15.5	27	46.8	14.8	46	55.2	15.2									
Education (years)	73	7.0	4.0	27	7.7	4.2	46	6.5	3.8									
**Neuropsychological tests**																		
Digit Span Forward	73	5.2	1.9	27	5.4	1.5	46	5.1	2.1	73	5.9	1.9	27	6.2	1.8	46	5.7	2.0
Digit Span Backward	73	3.4	1.8	27	3.2	1.7	46	3.5	1.8	73	3.8	1.7	27	3.7	1.8	46	3.9	1.7
Verbal Fluency Test	72	21.3	11.5	27	24.4	13.4	45	19.4	9.9	72	23.9	10.8	27	26.1	12.0	45	22.6	10.0
Stroop Dots	39	26.6	14.1	13	24.0	14.6	26	27.8	13.9	43	23.9	11.6	14	18.1	5.7	29	26.7	12.7
Stroop Color	38	50.9	29.8	13	48.8	37.9	25	52.0	25.3	43	56.4	35.7	14	43.3	29.4	29	62.8	37.3
Stroop Interference	38	0.5	0.4	13	0.7	0.4	25	0.4	0.3	43	0.6	0.4	14	0.8	0.4	29	0.6	0.3
**Clinical characteristics**																		
HAM-D-31	71	8.2	6.5	25	9.7	6.5	46	7.5	6.5	71	9.6	8.6	25	12.2	9.1	46	8.2	8.1
NIHSS	69	3.4	3.2	24	3.0	2.7	45	3.6	3.5	69	2.1	2.1	24	2.0	2.0	45	2.1	2.2
**Volume of stroke lesion**																		
Total cortex	51	2685.9	4058.2	23	2471.7	3751.4	28	2861.9	4354.4									
Dorsolateral prefrontal cortex	50	34.3	120.2	23	44.1	154.0	27	25.9	83.8									
Medial prefrontal cortex	50	102.8	235.4	23	69.1	168.5	27	131.5	280.3									
Orbital prefrontal cortex	50	49.0	167.7	23	18.0	56.5	27	75.4	220.8									

**Note:** Statistical analysis regarding the significance of differences between time assessments and between women and men is provided in Table 2. The Stroop test was included in the protocol during the study, and only 36 patients undertook it at the two timepoints. Volumes of stroke lesion in cortex areas are expressed in mm^3^, including the mean and standard deviation. Volume of stroke lesion in prefrontal cortex = BA10 (Brodmann area 10) + BA11 + BA12 + BA13 + BA47; volume of stroke lesion in medial prefrontal cortex = BA10 + BA11 + BA13 + BA14 + BA24 + BA25 + BA32 + BA47; volume of stroke lesion in dorsolateral prefrontal cortex = BA9; total volume of stroke lesion = sum of the volume of the 48 Brodmann areas. **Abbreviations:** T1 = first timepoint 12.0 (±3.8) days after stroke; T2 = second timepoint 91.6 (±5.4) days after stroke; HAM-D = Hamilton Rating Scale for Depression. 31-item version; NIHSS. National Institutes of Health Stroke Scale; SD = standard deviation; educational level = years of schooling; FAS test = verbal fluency test.

**Table 2:** Gender interaction effect for cognitive variables, severity of depressive symptoms, and functional status from the first to the fourth month after a first ischemic stroke.

	Time		Time × Gender		Gender	
Variables	*p*	ES	*p*	ES	*p*	ES
Digit Span Forward	0.001	0.031	0.723	0.000	0.347	0.010
Digit Span Backward	0.050	0.017	0.822	0.000	0.423	0.006
Verbal Fluency Test	0.005	0.011	0.415	0.001	0.108	0.033
Stroop Dots	<0.001	0.069	0.930	0.000	0.149	0.044
Stroop Color	0.487	0.002	0.412	0.002	0.509	0.011
Stroop Interference	0.011	0.042	0.637	0.001	0.061	0.070
HAM-D-31	0.194	0.010	0.481	0.003	0.031	0.038
NIHSS	<0.001	0.044	0.320	0.002	0.628	0.003

**Note:** η effect sizes for time, time × gender, and gender effects. Cohen’s d effect size for pairwise comparison. The Stroop test was included in the protocol during the study, and only 36 patients undertook it at the two timepoints. Interference = Stroop color (the time was adjusted by the number of correct answers) divided by Stroop dots (the time was adjusted by the number of correct answers). **Assessments were performed at two timepoints:** T1 = first timepoint; 12.0 (+3.8) days after stroke; T2 = second timepoint; 91.6 (+5.4) days after stroke; **ES** = effect size.

**Table 3:** Correlation of neuropsychological changes with changes in clinical variables from the first to the fourth month after a first ischemic stroke: Total sample and split by gender.

	T1									T2								
Variables	Total (N = 73)			Female (N = 27)			Male (N = 46)			Total (N = 73)			Female(N = 27)			Male (N = 46)		
	N	M	SD	N	M	SD	N	M	SD	N	M	SD	N	M	SD	N	M	SD
Age (years)	73	52.1	15.5	27	46.8	14.8	46	55.2	15.2									
Education (years)	73	7.0	4.0	27	7.7	4.2	46	6.5	3.8									
**Neuropsychological tests**																		
Digit Span Forward	73	5.2	1.9	27	5.4	1.5	46	5.1	2.1	73	5.9	1.9	27	6.2	1.8	46	5.7	2.0
Digit Span Backward	73	3.4	1.8	27	3.2	1.7	46	3.5	1.8	73	3.8	1.7	27	3.7	1.8	46	3.9	1.7
Verbal Fluency Test	72	21.3	11.5	27	24.4	13.4	45	19.4	9.9	72	23.9	10.8	27	26.1	12.0	45	22.6	10.0
Stroop Dots	39	26.6	14.1	13	24.0	14.6	26	27.8	13.9	43	23.9	11.6	14	18.1	5.7	29	26.7	12.7
Stroop Color	38	50.9	29.8	13	48.8	37.9	25	52.0	25.3	43	56.4	35.7	14	43.3	29.4	29	62.8	37.3
Stroop Interference	38	0.5	0.4	13	0.7	0.4	25	0.4	0.3	43	0.6	0.4	14	0.8	0.4	29	0.6	0.3
**Clinical characteristics**																		
HAM-D-31	71	8.2	6.5	25	9.7	6.5	46	7.5	6.5	71	9.6	8.6	25	12.2	9.1	46	8.2	8.1
NIHSS	69	3.4	3.2	24	3.0	2.7	45	3.6	3.5	69	2.1	2.1	24	2.0	2.0	45	2.1	2.2
**Volume of stroke lesion**																		
Total cortex	51	2685.9	4058.2	23	2471.7	3751.4	28	2861.9	4354.4									
Dorsolateral prefrontal cortex	50	34.3	120.2	23	44.1	154.0	27	25.9	83.8									
Medial prefrontal cortex	50	102.8	235.4	23	69.1	168.5	27	131.5	280.3									
Orbital prefrontal cortex	50	49.0	167.7	23	18.0	56.5	27	75.4	220.8									

**Note:** Spearman correlation coefficient. 2 = partial correlation controlled by age. *p*-Values are not adjusted for multiple comparisons. ∆ = T2 − T1 was calculated for all neuropsychological tests. **Stroop interference** = Stroop color (the time was adjusted by the number of correct answers) divided by Stroop dots (the time was adjusted by the number of correct answers). **Abbreviations:** T1 = first timepoint 12.0 (±3.8) days after stroke; T2 = second timepoint 91.6 (±5.4); Corr. Coeff. = Spearman’s correlation coefficient; HAM-D = Hamilton Rating Scale for Depression, 31-item version; NIHSS = National Institutes of Health Stroke Scale.

## Data Availability

Data is available by contacting the corresponding author.

## Supplementary Materials

The following supporting information can be downloaded at: https://www.mdpi.com/article/10.3390/life13071554/s1, Table S1: exploratory analysis of changes in cognitive performance from 1 to 4 months after stroke split by gender.
