# Supplementary material for: The Impact of Post-Stroke Depressive Symptoms on Cognitive Performance in Women and in Men: A 4 Month Prospective Study
Source: Life (Basel). 2023 Jul 13;13(7):1554. doi: 10.3390/life13071554 (PMC10381498; doi:10.3390/life13071554)
Supplement: Supplementary file 1 [file life-13-01554-s001.zip › life-2237377-supplementary.pdf]

The impact of post-stroke depressive symptoms on cognitive performance in women and in men: a four-month prospective study

Supplemental material

Table S1: exploratory analysis of changes in cognitive performance from 1 to 4 months after stroke split by gender

	Female X Male							
	Female		Male		T1		T2	
Variables	p	ES	p	ES	p	ES	p	ES
Digit Span Forward	**0.026**	0.453	**0.020**	0.474[1]	1.000	0.166[1]	0.650	0.246
Digit Span Backward	**0.040**	0.519[1]	0.155	0.271[1]	1.000	0.115[1]	1.000	0.043[1]
Verbal Fluency Test	0.302	0.203	**<0.001**	0.565	0.335	0.424	0.554	0.323
Stroop Dots	**0.026**	0.773[1]	**<0.001**	0.801[1]	0.581	0.311[1]	0.120	0.537[1]
Stroop Color	0.262	0.327	0.921	0.020	1.000	0.206[1]	1.000	0.357[1]
Stroop Interference	0.292	0.306	**0.004**	0.634	0.286	0.635	0.418	0.647[1]
HAM-D-31	0.299	0.212	0.570	0.084	0.687	0.249[1]	0.313	0.243[1]
NIHSS	**0.028**	0.579[1]	**<0.001**	0.787[1]	0.963	0.046[1]	0.963	0.064[1]

Note: *1 Nonparametric Rank Biserial correlation size effect.*
Abbreviations: T1=*First Time points****; T2=*** *Second time point; Cohen'd and Biserial effect size from pairwise comparison;* ***HAM-D =*** *Hamilton Rating Scale for Depression. 31-item version****; NIHSS****. National Institutes of Health Stroke Scale*
